# Tissue microarray analysis indicates hedgehog signaling as a potential prognostic factor in intermediate-risk prostate cancer

**DOI:** 10.1186/s12885-017-3619-4

**Published:** 2017-09-06

**Authors:** Annelies Gonnissen, Sofie Isebaert, Christiaan Perneel, Chad M. McKee, Clare Verrill, Richard J. Bryant, Filip Van Utterbeeck, Evelyne Lerut, Karin Haustermans, Ruth J. Muschel

**Affiliations:** 1 Department of Oncology, Laboratory of Experimental Radiotherapy, KU Leuven - University of Leuven, KU Leuven Campus Gasthuisberg, Herestraat 49, box 815, 3000 Leuven, Belgium; 2 Department of Radiation Oncology, University Hospitals Leuven, Leuven, Belgium; 3 Department of Applied Mathematics, Royal Military Academy, Brussels, Belgium; 4 Department of Oncology, CRUK/MRC Oxford Institute for Radiation Oncology, University of Oxford, Oxford, UK; 5 Nuffield Department of Surgical Sciences, University of Oxford, John Radcliffe Hospital, Oxford, UK; 6 Department of Pathology, University Hospitals Leuven, KU Leuven - University of Leuven, Leuven, Belgium

**Keywords:** Hedgehog pathway, Prostate cancer, Tissue microarray, Biochemical recurrence

## Abstract

**Background:**

Prostate cancer (PCa) is a heterogeneous disease with a variable natural history, genetics, and treatment outcome. The Hedgehog (Hh) signaling pathway is increasingly recognized as being potentially important for the development and progression of PCa. In this retrospective study, we compared the activation status of the Hh signaling pathway between benign and tumor tissue, and evaluated the clinical significance of Hh signaling in PCa.

**Methods:**

In this tissue microarray (TMA) study, the protein expression of several Hh signaling components and Hh target proteins, along with microvessel density, were compared between benign (*n* = 64) and malignant (*n* = 170) prostate tissue, and correlated with PCa clinicopathological characteristics and biochemical recurrence (BCR).

**Results:**

The Hh signaling pathway appeared to be more active in PCa than in benign prostate tissue, as demonstrated by lower expression of the negative regulators PTCH1 and GLI3 in the tumor tissue compared to benign. In addition, high epithelial GLI2 expression correlated with higher pathological Gleason score. Overall, higher epithelial GLI3 expression in the tumor was shown to be an independent marker of a favorable prognosis.

**Conclusion:**

Hh signaling activation might reflect aggressive tumoral behavior, since high epithelial GLI2 expression positively correlates with a higher pathological Gleason score. Moreover, higher epithelial GLI3 expression is an independent marker of a more favorable prognosis.

**Electronic supplementary material:**

The online version of this article (10.1186/s12885-017-3619-4) contains supplementary material, which is available to authorized users.

## Background

The Hedgehog (Hh) signaling pathway is an important developmental signaling pathway regulating cellular proliferation and differentiation, and tissue polarity, in several tissue types including the prostate gland during embryogenesis [1–3]. In normal adult tissues this pathway appears to be relatively quiescent, but it is important for maintenance of stem cell populations and for repair and regeneration following tissue damage [2, 4]. Reactivation of Hh signaling has recently been observed in multiple tumor types including prostate cancer (PCa) [2, 5].

**Fig. 1:**
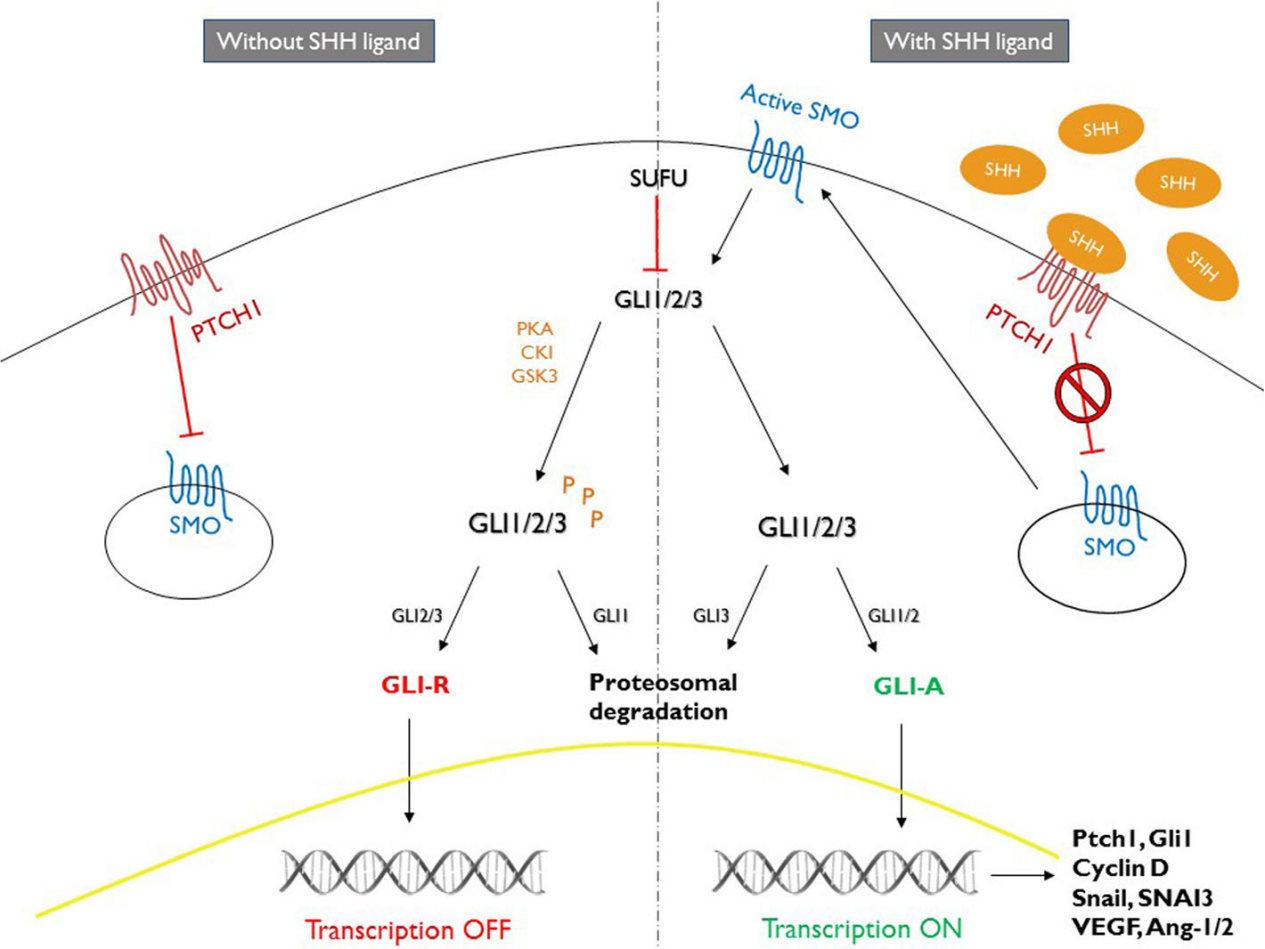
Schematic drawing illustrating the main components of the Hedgehog signaling cascade

Activation of the Hh signaling cascade is triggered by binding of a ligand such as Sonic Hedgehog (SHH) to the inhibitory receptor Patched 1 (PTCH1), which acts to alleviate the repression of Smoothened (SMO). In turn, SMO activates the downstream effectors of the Hh signaling cascade, including the Glioma-associated oncogene (GLI) transcription factors through inhibition of Suppressor of Fused (SUFU), which is a key negative regulator of Hh signaling [6, 7]. The GLI transcription factor family consists of three members, with GLI1 being the principle transcriptional activator. GLI2 has been shown to possess dual functionality, whilst GLI3 is primarily considered to be a Hh signaling repressor [8–10] (Fig. 1).

In this study the expression of Hh signaling components in benign and malignant prostate tissue was compared. PCa is a heterogeneous malignancy with a variable natural history, and the TMA cohort in this analysis is composed predominantly of intermediate-risk Gleason Sum score (GS) 7 PCa cases. It is a particular clinical challenge to tailor appropriate management of intermediate-risk PCa, and there is a limited number of clinically applicable biomarkers with which to risk-stratify intermediate-risk PCa according to its potential indolent or aggressive behaviour [11, 12]. Hh pathway protein expression was investigated in order to determine whether this has potential prognostic value in this category of PCa cases.

## Methods

### Tissue microarray

Tissue microarrays (TMAs) were constructed from formalin-fixed paraffin-embedded (FFPE) radical prostatectomy specimens from 170 PCa patients, and from trans-urethral resection of the prostate (TURP) samples from 64 patients with benign prostatic hyperplasia (BPH). The TMA consisted of a single tissue core for each of the 234 patients.

TMA sections were obtained from the Oxford Centre for Histopathology Research, Oxford University Hospitals NHS Foundation Trust, Oxford, UK. The TMA contained patient samples from Oxford and was built under the approval of the Oxford Radcliffe Biobank Ethics Committee (reference number 09/H0606/5 + 5). The study itself was approved by the ethics committee of KU Leuven, Leuven, Belgium (reference number S55726).

### Immunohistochemistry

Immunohistochemistry was performed using primary antibodies against SHH (1/50, Abcam ab53281), PTCH1 (1/300, Santa Cruz sc-6147), SMO (1/100, Abcam, ab72130), SUFU (1/100, Santa Cruz sc-28,847), GLI1 (1/50, Santa-Cruz sc-20,687), GLI2 (1/1000, Rockland 600–401-845), GLI3 (1/50, R&D AF3690), SNAIL (1/50, R&D, AF3639), SNAI3 (1/50, Novus Biologicals, NBP1–90661), CYCLIND1 (Dako, M364229) and CD31 (Dako, IR610 Clone JC70A). After incubation with the appropriate secondary antibody (Vector Laboratories), antigen presence was revealed with 3.3′-diaminobenzidine (DAB) substrate (Vector Laboratories, ImmPACT DAB, SK-4105) and slides were counterstained with hematoxylin. Control TURP resection specimens were used to validate the specificity of the primary antibodies, which was histopathologically assessed based on the expected subcellular localization of each protein and a lack of nonspecific background staining. For the validation of Snail expression, blood vessel staining was used as an internal positive control. To exclude any nonspecific staining of the secondary antibodies, negative controls were performed without the addition of any primary antibody.

### Immunohistochemistry scoring

Two independent researchers performed evaluation of the immunohistochemical staining for each protein, and each researcher was blinded to clinicopathological and outcome data. The percentage of stained cells (0–100%) and the staining intensity (0 = negative, 1 = weak, 2 = moderate, 3 = strong) were assessed both in the epithelial and stromal cells. In the malignant cores, only the tumoral glands were considered. Semi-quantitative analyses were performed by calculating histoscores (HS) as the product of the percentage stained cells (0–100) and staining intensity (0–3). If multiple staining intensities were present in the same core, the sum of the individual HS was taken to acquire the average HS of the entire core. Binary HS, with low and high expression respectively being defined as below and above the mean HS of 1.5, were used for statistical analyses. Microvessel density (MVD) was determined by counting the number of CD31-positive blood vessels in each core.

### Statistical analysis

The IBM/SPSS Statistics version 23/24 was used for statistical analyses. A Fisher’s exact test was used to compare the binary Hh expression level in the benign and malignant tissue cores, and to evaluate any potential association between the protein expression levels in the tumor and the clinicopathological parameters. One-way ANOVA with contrast analysis was used to assess any potential correlation between MVD and clinicopathological factors.

The impact of the studied proteins and clinicopathological factors on time to BCR was determined by a log-rank test and Kaplan-Meier analysis. A multivariate Cox proportional hazard regression model was used to determine the relative risk of important risk factors for BCR. Statistical results were considered significant at *p* < 0.05.

## Results

### PCa patient cohort characteristics

The patient and tumor characteristics of the 170 PCa patients are shown in Additional file 1: Table S1. The median age at the time of surgery was 61 years (range 45–71). Approximately two-thirds of patients had a pathological T stage (pT) ≤ pT2c, and only a small number of patients had a pathological GS ≥8. Median follow-up was 8.4 years (range 1.1–13.7), and 23% of patients developed biochemical recurrence (BCR) of PCa following radical surgery, as defined as a confirmed post-operative rise in PSA level to >0.2 ng/ml.

### Hh signaling in benign and cancerous prostate tissue

**Fig. 2:**
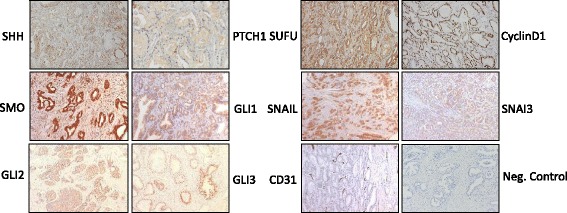
Representative images of positive IHC staining in benign and malignant prostate tissue and a negative control

Protein expression of the main Hh components in the PCa cores was compared with the expression level in the benign cores (Table 1 and Fig. 2). In general, Hh signaling protein expression was greater in the prostate epithelium than in the stromal tissue. When considering Hh signaling proteins in the epithelium, we observed that low levels of PTCH1 and GLI3 expression were more likely to occur in tumor tissue than in benign (PTCH1 76% PCa versus 26.6% benign, and GLI3 55.2% versus 29%, respectively, *p* < 0.001).

**Table 1:** Hh protein expression in benign and cancerous prostate tissue

	Benign prostate (n)		Prostate cancer (n)		Fisher’s exact test (2-sided)
Epithelial expression	Low (%)	High (%)	Low (%)	High (%)	*p*-value
SHH	46 (76.6)	14 (23.3)	111 (71.6)	44 (28.4)	0.497
PTCH1	17 (26.6)	47 (73.4)	114 (76)	36 (24)	*<0.001*
SMO	3 (4.7)	61 (95.3)	5 (3.2)	150 (96.8)	0.695
SUFU	19 (30.6)	43 (69.4)	26 (16.9)	128 (83.1)	*0.028*
GLI1	44 (73.3)	16 (26.7)	130 (83.9)	25 (16.1)	0.061
GLI2	5 (8.2)	56 (91.8)	54 (36.5)	94 (63.5)	*<0.001*
GLI3	18 (29)	44 (71)	85 (55.2)	69 (44.8)	*0.001*
Cyclin D1	54 (93.1)	4 (6.9)	94 (61.4)	59 (38.6)	*<0.001*
SNAIL	24 (39.3)	37 (60.7)	31 (20)	124 (80)	*0.005*
SNAI3	59 (95.2)	3 (4.8)	134 (86.5)	21 (13.5)	0.092
Stromal expression					
SHH	65 (100)	0	153 (100)	0	NA
PTCH1	65 (100)	0	152 (100)	0	NA
SMO	62 (95.4)	3 (4.6)	143 (94.7)	8 (5.3)	1.000
SUFU	23 (36.5)	40 (63.5)	25 (18.1)	113 (81.9)	*0.007*
GLI1	58 (93.5)	4 6.5)	141 (91.6)	13 (8.4)	0.783
GLI2	60 (93.8)	4 (6.3)	136 (95.1)	7 (4.9)	0.741
GLI3	63 (98.4)	1 (1.6)	146 (98)	3 (2)	1.000

Statistical significant results are presented in Italic

In contrast, high expression of the SUFU negative Hh regulator was observed in both benign and malignant prostate epithelium, with significantly higher levels being seen in tumors (*p* = 0.028).. GLI2 was observed to be widely and highly expressed in both benign and malignant prostate tissues. High levels of Cyclin D1 were more commonly seen in PCa than in benign prostate epithelium (*p* < 0.001). High epithelial SNAIL expression was also mainly observed in PCa compared with benign prostate epithelium (*p* = 0.005). Few differences in Hh signaling protein expression levels were observed in the stromal compartments of benign and malignant prostate tissue, with only SUFU being more highly expressed in the stromal compartment of PCa tissue than in benign cores (*p* < 0.007).

### Correlation between Hh signaling proteins and clinicopathological factors

We evaluated the clinical importance of Hh signaling protein expression in PCa patients. First, we investigated potential correlations between Hh protein expression and known prognostic clinicopathological factors such as pT stage, pathological GS, and surgical margin status. Higher epithelial GLI2 expression in the tumor was found to correlate with higher pathological GS (*p* = 0.047, Table 2), indicating that this might be a potential marker for aggressiveness in this PCa cohort. A trend was observed for higher epithelial GLI2 expression in tumors of a more advanced pT stage (*p* = 0.084).

**Table 2:** Correlation clinicopathological factors and epithelial GLI2 expression in the tumor

	Pathological T stage (n)			Pathological GS (n)			Surgical margin (n)		
Fisher’s exact test	pT ≤ 2 (%)	pT > 2 (%)	*p*-value	GS < 7 (%)	GS ≥ 7 (%)	*p*-value	Negative (%)	Positive (%)	*p*-value
Low epithelial GLI2	35 (64.8)	19 (35.2)	0.084	25 (46.3)	29 (53.7)	*0.047*	19 (35.2)	35 (64.8)	1.000
High epithelial GLI2	44 (48.9)	46 (51.1)		26 (28.9)	64 (71.1)		33 (36.7)	57 (63.3)	

*pT* pathological T stage; *GS* Gleason Score

Statistical significant results are presented in Italic

**Fig. 3:**
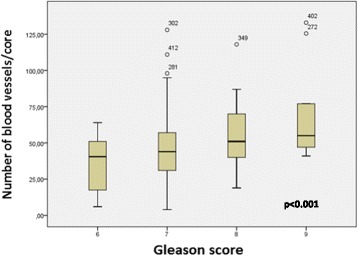
Microvessel density as a function of pathological Gleason score

We also observed a positive correlation between the mean vessel density (MVD) and pathological GS, suggesting that tumors with a more aggressive phenotype may present with a greater number of blood vessels (Fig. 3). The other Hh signaling proteins investigated did not demonstrate significant correlations with clinicopathological factors.

### Prognostic value of Hh signaling in prostate cancer

**Fig. 4:**
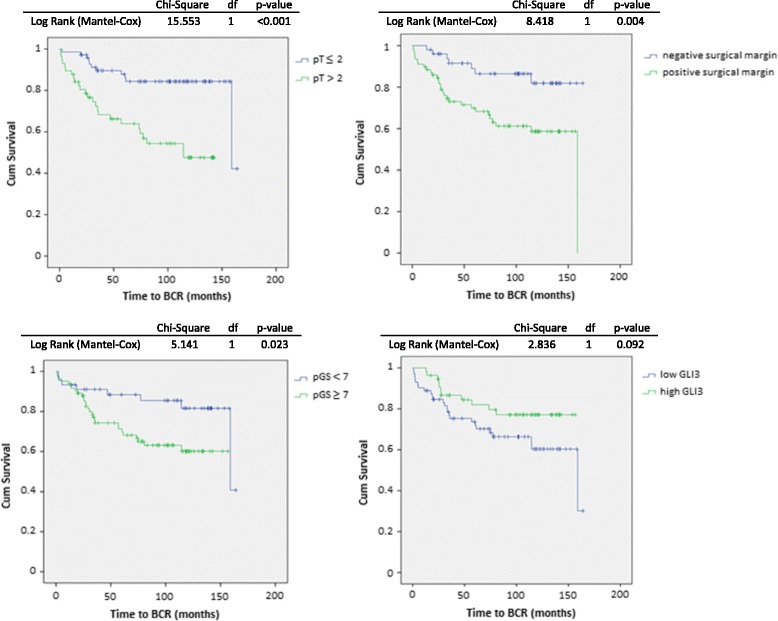
Survival analyses and log rank test for time to BCR according to pT stage (upper left), pGS (lower left), surgical margin (upper right) and epithelial GLI3 expression in the tumor (lower right)

Patients with a higher GS (pGS ≥ 7; *p* = 0.023), more advanced tumor stage (pT stage > pT2c; *p* < 0.001) and/or a positive surgical margin status (*p* = 0.004) had a shorter time to BCR where this occured. Higher epithelial GLI3 expression in the tumor showed a trend towards a better prognosis although this did not reach statistical significance in this cohort (*p* = 0.092) (Fig. 4).

In a multivariate Cox regression model, pT stage (HR = 3.317; *p* = 0.002), pathological GS (HR = 2.572; *p* = 0.4) and epithelial GLI3 expression (HR = 0.418; *p* = 0.023) were each found to be significant predictors of BCR (Table 3).

**Table 3:** Multivariate Cox regression model for BCR

	Hazard Ratio	95% CI	*p*-value
pT stage	3.317	1.562–7.045	*0.002*
Pathological GS	2.572	1.042–6.346	*0.04*
Epithelial GLI3	0.418	0.197–0.885	*0.023*

*pT* pathological T stage; *GS* Gleason score; *CI* Confidence interval

## Discussion

PCa is a heterogeneous disease with variable natural history, genetics, and treatment outcomes, and it is important to dissect the pathways that lead to its’ progression. This is particularly important in cases of intermediate-risk PCa, which will require the use of molecular tools in order to risk-stratify patients and better predict which men may have relatively indolent PCa which may not require immediate radical treatment with associated side-effects [13, 14]. There is therefore an important unmet clinical need to establish robust molecular markers with which to accurately predict disease progression. The Hh signaling pathway has been implicated as being one of the pathways that drive the PCa progression to a more advanced disease state. In this retrospective study we compared the activation status of the Hh pathway between benign and malignant prostate tissue, and evaluated the clinical significance of Hh signaling in a PCa.

Previous reports have demonstrated active Hh signaling in PCa tissue, with higher levels of expression of several Hh pathway components in PCa compared with benign prostate tissue [15, 16]. We similarly observed that Hh signaling appeared to be greater in malignant compared with benign prostate tissue, and in this cohort active Hh signaling status in PCa was mainly characterized by down-regulation of the PTCH1 and GLI3 negative regulators of this pathway. We also observed a positive correlation between high GLI2 expression in the tumor and a higher GS score, indicating that this might be a marker for an aggressive tumor phenotype. Narita et al. previously reported that GLI2 expression correlated with a more advanced PCa phenotype [17], whilst Kim et al. demonstrate correlations between Hh signaling molecules (GLI1, SHH, SMO, PTCH1) and GS score here, however in this latter study GLI2 expression was not assessed [18].

We observed that MVD was higher in PCa tumors with higher pathological GS score, indicating that more aggressive tumors have a greater degree of vascularization. This is consistent with a previous report by Erbersdobler et al. investigating a large PCa cohort [19], where MVD did not function as an independent prognostic marker, in line with our reported observations.

Our data demonstrated that pT stage and pathological GS are independent predictors of an adverse prognosis in terms of BCR following radical prostatectomy with curative intent. In addition, we demonstrate that epithelial GLI3 expression could represent a prognostic marker in surgical patients, with higher epithelial GLI3 expression reflecting a more favorable outcome. Other studies have previously suggested that the Hh signaling pathway might have a prognostic value in PCa [18, 20], and a study by McKee et al. demonstrated that Hh gene alterations are associated with an adverse prognosis in PCa patients [14].

## Conclusion

It is a particular clinical challenge to tailor appropriate management of intermediate-risk PCa, and there is only a limited number of clinically applicable biomarkers with which to risk-stratify these patients according to the potential indolent or aggressive nature of their disease. In this study, we demonstrated that active Hh signaling might reflect aggressive tumoral behavior, since high epithelial GLI2 expression positively correlates with a higher pathological GS. Moreover, higher epithelial GLI3 expression is an independent marker of a more favorable prognosis in this category of PCa cases.

## Additional file

Additional file 1: Table S1. Patient and tumor characteristics of PCa patients (*n* = 170). (DOCX 13 kb)

## Abbreviations

BCR: Biochemical recurrence
GLI: Glioma-associated oncogene
GS: Gleason score
GS: Gleason score
Hh: Hedgehog
HS: Histoscore
MVD: Microvessel density
PCa: Prostate cancer
pT: Pathological T stage
PTCH1: Patched 1
SHH: Sonic hedgehog
SMO: Smoothened
SUFU: Suppressor of fused
TMA: Tissue microarray
