# Supplementary material for: Tissue microarray analysis indicates hedgehog signaling as a potential prognostic factor in intermediate-risk prostate cancer
Source: BMC Cancer. 2017 Sep 6;17:634. doi: 10.1186/s12885-017-3619-4 (PMC5588741; doi:10.1186/s12885-017-3619-4)
Supplement: Additional file 1: Table S1. — Patient and tumor characteristics of PCa patients (n = 170). (DOCX 13 kb) [file 12885_2017_3619_MOESM1_ESM.docx]

| **Table S1. Patient and tumor characteristics (n=170)** | |
| --- | --- |
|  | **n** |
| **Median (range) age at surgery** | 61 years (45-71) |
| **Pathological T-stage**  2a  2b  2c  3a  3b  4  NA | 9 (5.3%)  14 (8.2%)  77 (45.3%)  58 (34.1%)  10 (5.9%)  1 (0.6%)  1 (0.6%) |
| **Pathological Gleason score**  5 (3+2)  6 (3+3)  7 (3+4)  7 (4+3)  8 (4+4)  9 (4+5)  NA | 2 (1.2%)  63 (37%)  74 (43.5%)  24 (14.1%)  4 (2.4%)  2 (1.2%)  1 (0.6%) |
| **Surgical margin**  Negative  Positive | 72 (42.4%)  98 (57.6%) |
| **Biochemical recurrence** | 39 (23%) |
| **Median (range) follow-up** | 8.4 years (1.1-13.7) |
